# Retinal Arteriosclerosis and Fundus Photograph-Defined Glaucoma in Young Adults: Findings from the Gangnam Eye Cohort

**DOI:** 10.3390/biomedicines14092059

**Published:** 2026-09-13

**Authors:** Eunoo Bak, Jin-Soo Kim, Dae Joong Ma, Jinho Lee, Baek Lok Oh, Hyuk Jin Choi

**Affiliations:** 1 Department of Ophthalmology, Uijeongbu Eulji Medical Center, Eulji University School of Medicine, Uijeongbu 11759, Republic of Korea; 2 Department of Ophthalmology, Seoul National University College of Medicine, Seoul 03080, Republic of Korea; 3 Department of Ophthalmology, Chungnam National University Sejong Hospital, Sejong 30099, Republic of Korea; 4 Department of Ophthalmology, Hallym University Kangnam Sacred Heart Hospital, Seoul 07441, Republic of Korea; 5 Department of Ophthalmology, Seoul National University Hospital Healthcare System Gangnam Center, Seoul 06236, Republic of Korea

**Keywords:** glaucoma, health screening cohort, retinal arteriosclerosis, young adult

## Abstract

**Background/Objectives**: To evaluate systemic and ocular factors associated with glaucoma in young adults using data from a large health screening cohort. **Methods**: This cross-sectional observational study analyzed baseline data from 16,002 Korean adults younger than 40 years (range, 18–39 years) in the Gangnam Eye Cohort. Participants underwent comprehensive health screening examinations, including standardized ophthalmic evaluation and fundus photography. Glaucoma was defined by structural glaucomatous changes on fundus photographs, and retinal arteriosclerosis was graded using a modified Scheie classification. Univariable and multivariable logistic regression models were used to identify factors associated with glaucoma. **Results**: Among 16,002 participants, 257 (1.61%) were classified as having glaucoma; 186 of these participants (72.4%) had unilateral glaucoma. In multivariable logistic regression analysis, retinal arteriosclerosis showed the strongest association with glaucoma (OR, 6.033; 95% CI, 4.417–8.240; *p* < 0.001). Older age, male sex, higher intraocular pressure, and higher low-density lipoprotein cholesterol level were also independently associated with glaucoma. A graded relationship was observed between retinal arteriosclerosis severity and glaucoma, with adjusted ORs of 5.51 (95% CI, 3.96–7.66) for grade 1 and 13.15 (95% CI, 6.41–26.99) for grade ≥ 2. **Conclusions**: In young adults, retinal arteriosclerosis was strongly and dose-dependently associated with glaucoma. These findings suggest that retinal microvascular abnormalities visible on routine fundus photographs may provide clinically accessible information for identifying young adults with structural features of glaucomatous optic neuropathy in health screening settings.

## 1. Introduction

Glaucoma is a progressive optic neuropathy characterized by the loss of retinal ganglion cells, leading to irreversible visual impairment and blindness worldwide [1]. Elevated intraocular pressure (IOP) is the most important modifiable risk factor and remains the primary target of current glaucoma treatment strategies [2,3]. However, glaucomatous damage can occur in individuals with normal or relatively low IOP, suggesting that mechanisms beyond IOP contribute to disease development and progression [4,5,6]. Among these mechanisms, vascular dysfunction has been increasingly implicated in glaucomatous optic neuropathy, with impaired ocular perfusion and abnormal vascular autoregulation proposed as potential contributors [7,8,9].

Although numerous epidemiologic studies have investigated risk factors for glaucoma, most have focused on middle-aged or elderly populations in whom the disease is more prevalent [8,10,11]. In contrast, glaucoma in young adults is relatively uncommon [12,13], and the factors associated with early glaucomatous damage in this age group remain poorly understood. Identifying risk factors in younger individuals may provide insight into early disease mechanisms and help detect individuals at higher risk before significant structural or functional damage occurs. Large health screening cohorts provide a unique opportunity to investigate systemic and ocular factors associated with glaucoma in relatively young populations using standardized clinical and laboratory data.

Therefore, the purpose of this study was to evaluate systemic and ocular factors associated with glaucoma in adults younger than 40 years using data from a large health screening cohort. We specifically examined whether retinal arteriosclerosis, a clinically recognizable marker of retinal microvascular alteration, was associated with fundus photograph–defined glaucoma after accounting for conventional ocular and systemic factors.

## 2. Materials and Methods

### 2.1. Study Design and Population

This cross-sectional study used baseline data from the Gangnam Eye Cohort, an ongoing health screening centre–based retrospective ophthalmic cohort conducted at the Seoul National University Hospital Healthcare System Gangnam Centre in Seoul, Republic of Korea. The Gangnam Centre is a large health screening facility in which participants voluntarily undergo repeated comprehensive health examinations, typically at yearly intervals. For the present study, we used baseline data from the Gangnam Eye Cohort. Among 76,030 individuals who underwent health screening between October 2003 and December 2010, 496 non-Korean participants were excluded, leaving 75,534 Korean participants. A further 380 participants were excluded because of poor-quality fundus photographs, pathologic myopia, or abnormal disc morphology, resulting in 75,154 eligible participants, as previously described [14].

Of these 75,154 participants, we included young adults, defined as individuals aged 18 to 39 years, consistent with previous ophthalmic studies examining adults younger than 40 years [15,16,17]. Analyses were conducted at the participant level. Participants were classified according to the study glaucoma coding system, and only those classified as normal or glaucoma were included in the primary analysis; glaucoma suspect cases were excluded. For ocular variables, the glaucomatous eye was selected in participants with unilateral glaucoma. In participants with bilateral glaucoma or bilateral normal eyes, one eye was randomly selected for analysis.

This study was approved by the Institutional Review Board of Seoul National University Hospital (IRB No. H-1906-141-1043) and adhered to the tenets of the Declaration of Helsinki. The requirement for informed consent was waived by the Institutional Review Board owing to the retrospective nature of the study.

### 2.2. Comprehensive Health Screening Examinations

The health screening examination consisted of a standardized health interview survey and a health examination survey including ophthalmic examinations. The health interview survey collected information on demographic characteristics, medical history, health-related behaviours, and socioeconomic variables. Medical history variables included diabetes mellitus, hypertension, dyslipidemia, coronary heart disease, and metabolic syndrome. Metabolic syndrome was defined according to previously described criteria [18]. Smoking status was categorized as never, former, or current smoker, and alcohol consumption was categorized as never, former, or current drinker. Household income was categorized as <5 million Korean won (KRW)/month or ≥5 million KRW/month. Educational attainment was categorized according to whether participants had completed college or higher education.

The health examination survey included anthropometric measurements and laboratory testing. Height, weight, waist circumference, and blood pressure were measured by trained nurses, and body mass index (BMI) was calculated as weight in kilograms divided by height in metres squared. Abnormal waist circumference was defined as ≥90 cm in men and ≥85 cm in women. Laboratory measurements included fasting blood glucose, glycated hemoglobin, lipid profiles, renal function markers, liver function markers, inflammatory markers, complete blood count parameters, apolipoproteins, and serologic markers. These variables were obtained using standardized protocols used in the Gangnam Eye Cohort [14].

### 2.3. Ophthalmic Examination

The ophthalmic screening examination included visual acuity testing, intraocular pressure (IOP) measurement using a noncontact tonometer (CT-80; Topcon Inc., Tokyo, Japan), and nonmydriatic fundus photography using a 45-degree field digital fundus camera (CR6-45NW; Canon Inc., Tokyo, Japan) in a dark room. During the study period, fundus photography was routinely performed in participants aged 35 years or older, whereas participants younger than 35 years generally underwent fundus photography upon request. Retinal arteriosclerosis was graded from fundus photographs using Scheie’s classification system with slight modification. Grade 0 indicates no apparent change; grade 1, mild arteriolar narrowing with increased light reflex; grade 2, obvious arteriolar narrowing with focal irregularities, a prominent light reflex, and the presence of arteriovenous crossing change; and grade 3, advanced arteriosclerosis characterized by copper- or silver-wire appearance with more pronounced arteriovenous crossing signs. Retinal arteriosclerosis was independently graded by two retinal specialists, separately from glaucoma grading performed by glaucoma specialists, with disagreements resolved by consensus. The retinal and glaucoma grading groups were mutually masked to each other’s grading results. Retinal arteriosclerosis was analyzed both as a binary variable, defined as grade ≥ 1, and as a severity variable categorized as grade 0, grade 1, and grade ≥ 2 because few participants had grade 3 disease [19,20].

### 2.4. Definition of Glaucoma

In this screening cohort, glaucoma classification was based on structural optic disc and retinal nerve fibre layer (RNFL) changes identified on fundus photographs according to the Gangnam Eye Cohort grading protocol [14]. This structural classification did not incorporate intraocular pressure or visual field findings and was based on predefined fundus photograph criteria consistent with previously published fundus photograph–based glaucoma classification systems [21,22]. Fundus photographs were independently evaluated by three glaucoma specialists who were masked to other ocular and systemic information. Vertical cup-to-disc ratio and neuroretinal rim width were measured on disc photographs by a glaucoma specialist and independently reviewed by the other examiners, who also assessed the presence of RNFL defects. An RNFL defect was considered suggestive of glaucoma when its width at the disc margin was larger than a major retinal vessel and diverged in an arcuate or wedge-shaped pattern. When classifications were discordant, consensus was reached through discussion with a fourth glaucoma specialist.

Eyes were classified as glaucoma if they had a vertical cup-to-disc ratio ≥ 0.9, neuroretinal rim width ≤ 0.05 of the disc diameter, an RNFL defect compatible with optic disc appearance, or optic disc hemorrhage corresponding to an RNFL defect. Eyes classified as glaucoma suspects, including those with vertical cup-to-disc ratio ≥ 0.7 without RNFL defect, rim width ≤ 0.1 of the disc diameter without RNFL defect, optic disc hemorrhage without RNFL defect, or RNFL defect without glaucomatous optic disc change, were excluded from the present analysis. For participant-level classification, individuals were considered to have glaucoma if glaucoma was identified in at least one eligible eye. Because this study was conducted in a health screening setting, standard automated perimetry was not routinely available for all participants.

### 2.5. Statistical Analysis

Baseline characteristics were compared between participants with and without glaucoma using Student’s *t*-test for continuous variables and the chi-square test for categorical variables. Factors associated with glaucoma were evaluated using participant-level logistic regression. Univariable logistic regression analyses were performed for demographic, socioeconomic, medical history, lifestyle, anthropometric, ocular, and laboratory variables. Factors associated with glaucoma were evaluated using participant-level multivariable logistic regression. The multivariable model included retinal arteriosclerosis, intraocular pressure, age, sex, body mass index, smoking status, alcohol consumption, systolic blood pressure, lipid parameters, glucose, renal-metabolic laboratory markers, and liver enzymes. Covariates were selected a priori based on clinical relevance and prior epidemiologic evidence. Variables representing overlapping clinical constructs were not included simultaneously to reduce multicollinearity. Multicollinearity was assessed using variance inflation factors; no covariate had a variance inflation factor > 10, and all prespecified variables were retained in the final model. Odds ratios (ORs) and 95% confidence intervals (CIs) were calculated. A two-sided *p* value < 0.05 was considered statistically significant. Statistical analyses were performed using SAS version 9.4 (SAS Institute, Cary, NC, USA).

## 3. Results

### 3.1. Study Population

A total of 16,002 participants younger than 40 years were included in the analysis. Of these, 257 participants (1.61%) were classified as having glaucoma based on structural glaucomatous changes identified on fundus photographs, including 176 men and 81 women; 186 participants (72.4%) had unilateral glaucoma, and 71 participants (27.6%) had bilateral glaucoma. The mean age of the study population was 35.30 ± 3.29 years. Of the 16,002 participants, 4876 (30.47%) were aged 18–34 years and 11,126 (69.53%) were aged 35–39 years (Table S1). Baseline characteristics according to glaucoma status are summarized in Table 1. Participants with glaucoma were older and were more frequently male, ever smokers, and current or past drinkers; and had higher body mass index, blood pressure, LDL cholesterol, triglycerides, creatinine, uric acid, and intraocular pressure. Retinal arteriosclerosis was also more prevalent in participants with glaucoma. Detailed comparisons of additional baseline characteristics are presented in Table S2.

### 3.2. Factors Associated with Glaucoma

Univariable logistic regression analyses for demographic, systemic, ocular, and laboratory factors are presented in Table S3. In multivariable logistic regression analysis (Table 2), older age (OR, 1.053 per year; 95% CI, 1.004–1.104; *p* = 0.035) and male sex (OR, 1.981; 95% CI, 1.212–3.240; *p* = 0.006) were independently associated with glaucoma. Among ocular variables, higher intraocular pressure was associated with increased odds of glaucoma (OR, 1.151 per mm Hg; 95% CI, 1.099–1.205; *p* < 0.001), and retinal arteriosclerosis showed the strongest association (OR, 6.033; 95% CI, 4.417–8.240; *p* < 0.001). Among systemic variables, higher LDL cholesterol level was modestly associated with glaucoma (OR, 1.004 per 10 mg/dL; 95% CI, 1.000–1.009; *p* = 0.039).

### 3.3. Retinal Arteriosclerosis Severity and Glaucoma Risk

**Figure 1:**
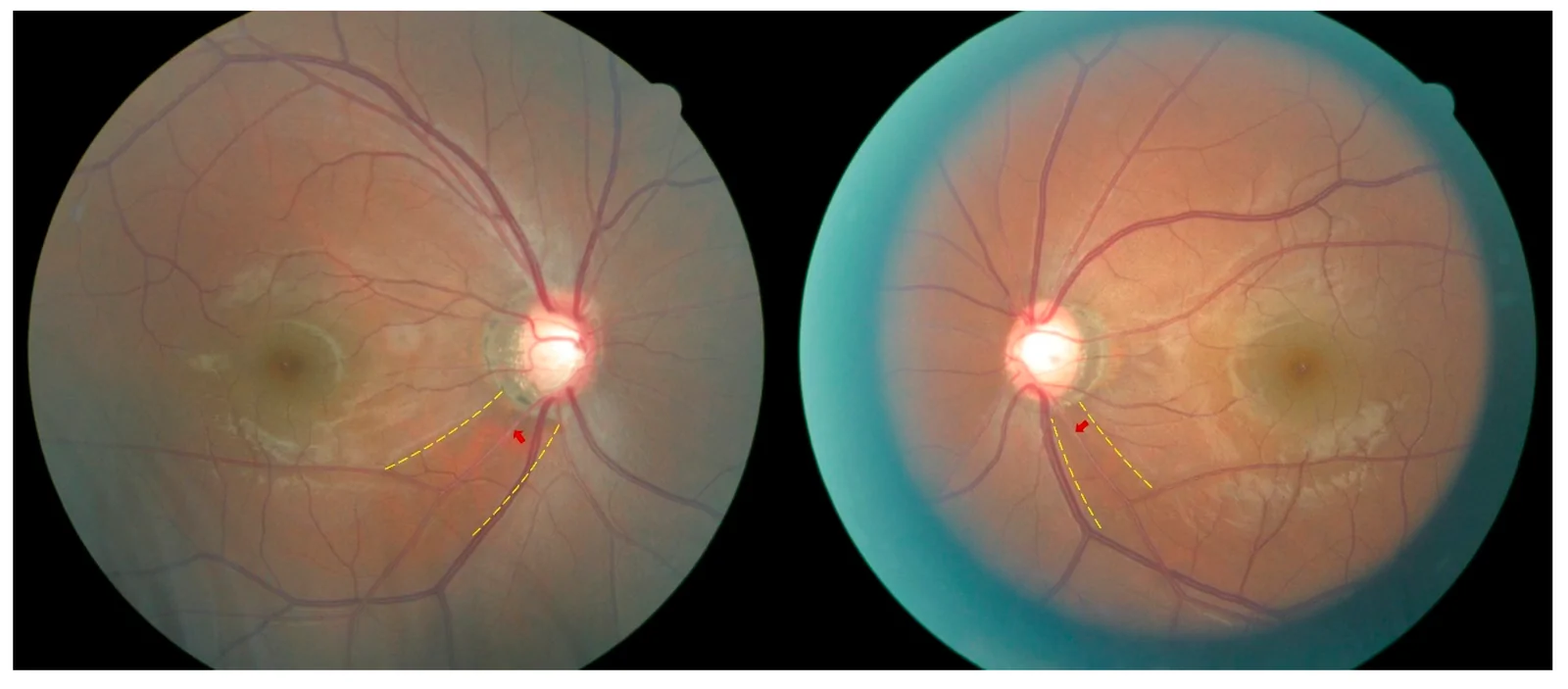
Representative Fundus Photographs of Bilateral Fundus Photograph–Defined Glaucoma with Retinal Arteriosclerosis. Representative fundus photographs of a 36-year-old man with bilateral fundus photograph–defined glaucoma and retinal arteriosclerosis. Yellow dashed lines indicate localized retinal nerve fibre layer (RNFL) defects, and red arrows indicate retinal arteriolar sclerotic changes (modified Scheie grade 2). Both eyes demonstrate enlarged glaucomatous optic disc cupping with localized RNFL defects. Glaucoma classification was based on glaucomatous optic disc and RNFL changes on fundus photographs and was performed independently of retinal arteriosclerosis grading.

A significant dose–response relationship was observed between the severity of retinal arteriosclerosis and glaucoma prevalence (Table 3). The prevalence of glaucoma increased progressively from 1.26% among participants without retinal arteriosclerosis to 7.07% in those with grade 1 and 13.89% in those with grade ≥ 2 changes. This trend remained evident after adjustment for confounders; compared with participants without retinal arteriosclerosis, the adjusted odds for glaucoma were 5.51 (95% CI, 3.96–7.66) for grade 1 and 13.15 (95% CI, 6.41–26.99) for grade ≥ 2 (both *p* < 0.001). Representative fundus photographs of a participant with bilateral glaucoma and retinal arteriosclerosis are shown in Figure 1.

## 4. Discussion

In this large health screening cohort of young adults, we identified several systemic and ocular factors associated with glaucoma. Retinal arteriosclerosis showed a significant association with glaucoma, and the prevalence of glaucoma increased with increasing severity of arteriosclerosis. In addition, demographic and systemic variables including increasing age, male sex, and higher serum LDL cholesterol levels demonstrated modest associations with glaucoma. These findings suggest that retinal microvascular alterations may be relevant to structural glaucomatous optic neuropathy even in relatively young individuals.

Most epidemiologic studies evaluating glaucoma risk factors have primarily focused on middle-aged or elderly populations because the prevalence of glaucoma increases substantially with age [1,23,24]. Consequently, relatively little is known about factors associated with glaucoma in younger adults [12,13]. In the present study, glaucoma was identified in 1.61% of participants, which is higher than the 0.16% to 0.4% prevalence reported in previous studies of adults aged 18 to 40 years [13]. In addition, 72.4% of affected participants had unilateral disease. This higher estimate may partly reflect the structural, fundus photograph–based definition used in the present screening cohort, which may identify early or preperimetric glaucomatous optic nerve changes that would not necessarily meet clinically confirmed glaucoma definitions requiring visual field loss.

Our findings indicate that even among adults younger than 40 years, increasing age remained associated with glaucoma, which is consistent with the well-established relationship between age and glaucomatous optic neuropathy [1,12,25,26]. The association observed with male sex has been reported in some population-based studies, although sex differences in glaucoma prevalence have not been entirely consistent across different cohorts [13,14,25,27]. These findings suggest that conventional glaucoma-related factors, including age and sex, may remain relevant even in younger adult populations.

The association between retinal arteriosclerosis and glaucoma observed in this study is particularly noteworthy. Our previous baseline report from the Gangnam Eye Cohort, which included a broader adult population, similarly identified older age, higher IOP, and retinal arteriosclerosis as factors associated with glaucoma [14]. The present study extends these findings by focusing specifically on young adults and demonstrating a graded association between retinal arteriosclerosis severity and glaucoma prevalence. Retinal arteriosclerosis reflects structural changes in retinal arteriolar walls that are generally considered manifestations of microvascular damage and vascular ageing [28,29,30,31]. The retinal vasculature shares anatomical and physiological characteristics with the circulation supplying the optic nerve head, and alterations in retinal microvasculature may therefore reflect compromised ocular perfusion or vascular dysregulation that could contribute to glaucomatous optic nerve damage [32]. Impaired ocular blood flow and vascular dysregulation have been proposed as potential mechanisms contributing to glaucomatous optic neuropathy, particularly in cases where intraocular pressure alone does not fully explain disease occurrence [7]. The graded relationship observed between the severity of retinal arteriosclerosis and glaucoma prevalence in this study further supports the possibility that retinal microvascular alterations detectable on routine fundus examination may reflect vascular conditions relevant to glaucomatous optic nerve damage.

In addition to retinal vascular findings, systemic and ocular factors showed modest associations with glaucoma in this relatively young population. Higher IOP was independently associated with glaucoma, consistent with its well-established role in glaucomatous optic neuropathy [3]. We also observed an association between LDL cholesterol level and glaucoma; however, this finding should be interpreted cautiously because the effect size was small and the odds ratio was close to unity. Although dyslipidemia has been implicated in systemic vascular dysfunction and atherosclerotic disease, its role in glaucoma remains uncertain, and epidemiologic studies have reported inconsistent findings regarding the association between serum lipid levels and glaucoma [33,34]. Therefore, although the LDL cholesterol finding may be consistent with a broader vascular-metabolic hypothesis, it should be regarded as exploratory rather than definitive. Further longitudinal studies are needed to clarify whether dyslipidemia contributes independently to glaucomatous optic nerve damage or merely reflects underlying vascular risk profiles associated with glaucoma.

Several limitations of this study should be considered. First, the diagnosis of glaucoma in this health screening cohort was based on fundus photograph evaluation rather than comprehensive clinical examinations including standard automated perimetry. Because visual field testing is not routinely performed as part of general health screening programmes, functional confirmation of glaucomatous visual field loss was not available. Accordingly, the outcome in this study should be interpreted as fundus photograph-defined glaucoma rather than a comprehensive clinical diagnosis incorporating functional assessment. However, structural optic disc and retinal nerve fibre layer changes identified on fundus photographs are commonly used to identify eyes with features suggestive of glaucoma that may warrant referral for further evaluation in screening settings [13]. Second, although the study included participants aged 18 to 39 years, the age distribution was weighed toward the older portion of this range, with 69.5% of participants aged 35 to 39 years, because fundus photography was routinely performed in participants aged 35 years or older but only selectively in younger participants. Therefore, the findings may not be fully generalized to younger adults, particularly those younger than 35 years. Third, the cross-sectional nature of this study limits the ability to determine causal relationships between retinal arteriosclerosis and glaucoma. Longitudinal studies are needed to clarify whether retinal vascular changes precede the development or progression of glaucomatous damage. Fourth, detailed ocular imaging of retinal microvasculature, such as optical coherence tomography angiography (OCTA), was not available in this screening-based dataset. Future studies incorporating OCTA may help further elucidate the relationship between retinal microvascular alterations and glaucomatous optic neuropathy [35,36].

In conclusion, in this health screening cohort of young adults, retinal arteriosclerosis was significantly associated with glaucoma, with a graded relationship between arteriosclerosis severity and glaucoma prevalence. These findings suggest that retinal microvascular changes may be relevant to glaucomatous optic nerve damage even in relatively young individuals and may provide clinically accessible information when evaluating glaucoma risk in screening settings.

## Figures and Tables

**Table 1:** Baseline Characteristics of Participants According to Glaucoma Status.

Variable	Normal (n = 15,745)	Glaucoma (n = 257)	*p* Value
**Demographics**			
Age (y)	35.30 ± 3.29	35.87 ± 2.93	0.002 ^b^
Male sex, n (%)	7964 (50.6)	176 (68.5)	<0.001 ^a^
**Medical history**			
Diabetes mellitus, n (%)	485 (3.1)	8 (3.1)	0.97 ^a^
Hypertension, n (%)	1456 (9.2)	38 (14.8)	0.002 ^a^
Hyperlipidemia, n (%)	2134 (13.6)	42 (16.3)	0.19 ^a^
Heart disease, n (%)	245 (1.6)	5 (1.9)	0.61 ^a^
Metabolic syndrome, n (%)	2038 (12.9)	43 (16.7)	0.07 ^a^
**Health-related behaviours**			
Smoking status, n (%)			0.049 ^a^
No	8245 (52.4)	119 (46.3)	
Past	2819 (17.9)	61 (23.7)	
Current	4268 (27.1)	71 (27.6)	
Drinking status, n (%)			0.040 ^a^
No	3288 (20.9)	37 (14.4)	
Past	623 (4.0)	9 (3.5)	
Current	11,413 (72.5)	202 (78.6)	
**Socioeconomic factors**			
Household income, n (%)			0.003 ^a^
<5 million KRW/month	6695 (42.5)	90 (35.0)	
≥5 million KRW/month	7990 (50.7)	155 (60.3)	
Education, n (%)			0.015 ^a^
<College	2700 (17.1)	28 (10.9)	
≥College	12,820 (81.4)	225 (87.5)	
**Anthropometric measurements**			
BMI (kg/m^2^)	22.78 ± 3.51	23.38 ± 3.27	0.004 ^b^
WC (cm), n (%)			0.26 ^a^
Normal	12,527 (79.6)	195 (75.9)	
Abnormal	3144 (20.0)	59 (23.0)	
Blood pressure (mm Hg)			
SBP, mm Hg	112.43 ± 13.88	116.00 ± 14.04	<0.001 ^b^
DBP, mm Hg	72.43 ± 11.42	75.38 ± 11.45	<0.001 ^b^
**Ocular characteristics**			
IOP (mm Hg)	13.43 ± 2.77	14.75 ± 3.54	<0.001 ^b^
Retinal arteriosclerosis, n (%)			<0.001
Absent (Grade 0)	14,934 (94.8)	190 (73.9)	
Present	811 (5.2)	67 (26.1)	
Grade 1	749 (4.8)	57 (22.2)	
Grade 2	58 (0.4)	10 (3.9)	
Grade 3	4 (0.03)	0 (0.0)	
**Laboratory data**			
Glucose, mg/dL	92.82 ± 14.20	93.27 ± 11.80	0.61 ^b^
Creatinine, mg/dL	0.98 ± 0.18	1.02 ± 0.18	<0.001 ^b^
AST, U/L	22.40 ± 15.32	22.62 ± 9.21	0.82 ^b^
ALT, U/L	25.49 ± 30.12	26.89 ± 20.92	0.46 ^b^
GGT, U/L	31.40 ± 40.47	34.50 ± 37.78	0.22 ^b^
Total bilirubin, mg/dL	1.09 ± 0.68	1.10 ± 0.41	0.80 ^b^
Uric acid, mg/dL	5.47 ± 1.56	5.91 ± 1.54	<0.001 ^b^
LDL cholesterol, mg/dL	110.59 ± 30.31	119.00 ± 33.39	<0.001 ^b^
HDL, mg/dL	55.30 ± 13.47	52.31 ± 12.32	<0.001 ^b^
Triglycerides, mg/dL	109.37 ± 85.35	122.58 ± 76.08	0.013 ^b^
Albumin, g/dL	4.47 ± 0.25	4.51 ± 0.25	0.019 ^b^
Hs-CRP	0.11 ± 0.32	0.14 ± 0.31	0.28 ^b^

Data are presented as mean ± SD or number (%). ^a^ *p* values were calculated using the χ^2^ test. ^b^ *p* values were calculated using the Student *t* test. ALT = alanine aminotransferase; AST = aspartate aminotransferase; BMI = body mass index; BUN = blood urea nitrogen; DBP = diastolic blood pressure; GGT = gamma-glutamyl transferase; HDL = high-density lipoprotein; Hs-CRP = high-sensitivity C-reactive protein; IOP = intraocular pressure; LDL = low-density lipoprotein; SBP = systolic blood pressure.

**Table 2:** Multivariable Logistic Regression Analysis of Factors Associated with Glaucoma.

Variable	Odds Ratio (95% CI)	*p* Value
Age, per year	1.053 (1.004–1.104)	**0.035**
Sex, male vs. female	1.981 (1.212–3.240)	**0.006**
Smoking, yes vs. no	0.859 (0.638–1.156)	0.316
Drinking, yes vs. no	1.350 (0.915–1.993)	0.13
BMI, per kg/m^2^	0.950 (0.901–1.002)	0.061
IOP, per mm Hg	1.151 (1.099–1.205)	**<0.001**
Retinal arteriosclerosis, grade ≥ 1	6.033 (4.417–8.240)	**<0.001**
SBP, per mm Hg	0.999 (0.988–1.010)	0.829
LDL cholesterol, per 10 mg/dL	1.004 (1.000–1.009)	**0.039**
HDL cholesterol, per 10 mg/dL	0.990 (0.977–1.003)	0.116
Triglycerides, per 10 mg/dL	1.000 (0.998–1.002)	0.946
Glucose, per mg/dL	0.990 (0.978–1.002)	0.089
Creatinine, per mg/dL	0.817 (0.289–2.312)	0.704
Uric acid, per mg/dL	1.054 (0.938–1.186)	0.376
Albumin, per g/dL	1.093 (0.599–1.994)	0.772
Total bilirubin, per mg/dL	0.836 (0.608–1.151)	0.273
AST, per U/L	1.000 (0.985–1.016)	0.973
ALT, per U/L	0.997 (0.986–1.007)	0.541
GGT, per U/L	0.999 (0.994–1.004)	0.623

ALT = alanine aminotransferase; AST = aspartate aminotransferase; BMI = body mass index; GGT = gamma-glutamyl transferase; HDL = high-density lipoprotein; IOP = intraocular pressure; LDL = low-density lipoprotein; SBP = systolic blood pressure. Statistically significant values are shown in boldface type.

**Table 3:** Association Between Retinal Arteriosclerosis Severity and Glaucoma.

Grade	Participants, N	Glaucoma, N	Glaucoma Prevalence, %	Adjusted OR (95% CI) vs. Grade 0	*p* Value
0	15,124	190	1.26	1 [Reference]	—
1	806	57	7.07	5.51 (3.96–7.66)	<0.001
≥2	72	10	13.89	13.15 (6.41–26.99)	<0.001

The multivariable model included age, sex, smoking status, alcohol consumption, body mass index, intraocular pressure, systolic blood pressure, LDL cholesterol, HDL cholesterol, triglycerides, glucose, creatinine, uric acid, total bilirubin, AST, ALT, and GGT. Abbreviations: CI = confidence interval; OR = odds ratio.

## Data Availability

The data presented in this study are available from the corresponding author upon request. The data are not publicly available due to privacy and ethical restrictions.

## Supplementary Materials

The following supporting information can be downloaded at https://www.mdpi.com/article/10.3390/biomedicines14092059/s1, Table S1: Distribution of Study Participants and Glaucoma Prevalence According to Age Group; Table S2: Detailed Baseline Systemic Characteristics of Study Population; Table S3: Univariable Logistic Regression Analysis of Factors Associated with Glaucoma.

## Abbreviations

The following abbreviations are used in this manuscript:

BMI	body mass index
CI	confidence interval
HDL	high-density lipoprotein
IOP	intraocular pressure
KRW	Korean won
LDL	low-density lipoprotein
OCTA	optical coherence tomography angiography
OR	odds ratio
RNFL	retinal nerve fibre layer
