# Supplementary material for: Retinal Arteriosclerosis and Fundus Photograph-Defined Glaucoma in Young Adults: Findings from the Gangnam Eye Cohort
Source: Biomedicines. 2026 Sep 13;14(9):2059. doi: 10.3390/biomedicines14092059 (PMC13604444; doi:10.3390/biomedicines14092059)
Supplement: Supplementary file 1 [file biomedicines-14-02059-s001.zip › biomedicines-4496739-supplementary.pdf]

Table S1. Distribution of Study Participants and Glaucoma Prevalence According to Age Group.

Age group, years	Participants, N (%)	Glaucoma, N (%)
18–24	182 (1.14)	2 (1.10)
25–29	803 (5.02)	6 (0.75)
30–34	3891 (24.32)	49 (1.26)
35–39	11,126 (69.53)	200 (1.80)
Total	16,002 (100)	257 (1.61)

Percentages in the “Participants” column represent the proportion of the total study population, whereas percentages in the “Glaucoma” column represent glaucoma prevalence within each age group.

Table S2. Detailed Baseline Systemic Characteristics of Study Population.

Variable	Normal (n=15,745)	Glaucoma (n=257)	*p* Value
**Blood test**			
Glucose, mg/dL	92.82±14.20	93.27±11.80	0.61[b]
HbA1c, %	5.52±0.52	5.49±0.59	0.41[b]
Ca, mg/dL	9.25±0.38	9.29±0.39	0.07[b]
P, mM/L	3.60±0.48	3.56±0.47	0.13[b]
BUN, mg/dL	12.36±3.12	12.42±2.88	0.76[b]
Creatinine, mg/dL	0.98±0.18	1.02±0.18	<0.001[b]
AST, U/L	22.40±15.32	22.62±9.21	0.82[b]
ALT, U/L	25.49±30.12	26.89±20.92	0.46[b]
ALP, IU/L	57.16±16.89	58.65±18.52	0.16[b]
GGT, U/L	31.40±40.47	34.50±37.78	0.22[b]
Total bilirubin, mg/dL	1.09±0.68	1.10±0.41	0.80[b]
Uric acid, mg/dL	5.47±1.56	5.91±1.54	<0.001[b]
Total cholesterol, mg/dL	185.57±32.71	192.08±35.50	0.002[b]
LDL cholesterol, mg/dL	110.59±30.31	119.00±33.39	<0.001[b]
HDL, mg/dL	55.30±13.47	52.31±12.32	<0.001[b]
Triglycerides, mg/dL	109.37±85.35	122.58±76.08	0.013[b]
Total protein, g/dL	7.26±0.40	7.24±0.41	0.46[b]
Albumin, g/dL	4.47±0.25	4.51±0.25	0.019[b]
Hs-CRP	0.11±0.32	0.14±0.31	0.28[b]
Blood concentration of			
WBC, $\times 10^3/\mu L$	5.86±2.32	5.92±1.74	0.07[b]
RBC, $\times 10^3/\mu L$	4.70±0.49	4.86±0.48	0.012[b]
Hematocrit, %	42.78±4.51	43.88±4.23	0.011[b]
HBsAg, n (%)	632 (4.0)	10 (3.9)	0.92[a]
HCV Ab, n (%)	73 (0.5)	1 (0.4)	0.87[a]

Data are presented as mean ± SD or number (%). [a] *p* values were calculated using the $\chi 2$ test. [b] *p* values were calculated using the Student *t* test. ALP = alkaline phosphatase; ALT = alanine aminotransferase; AST = aspartate aminotransferase; BUN = blood urea nitrogen; Ca = calcium; GGT = gamma-glutamyl transferase; HbA1c = hemoglobin A1c; HBsAg = hepatitis B surface antigen; HCV-Ab = hepatitis C virus antibody; HDL = high-density lipoprotein; Hs-CRP = high-sensitivity C-

reactive protein; LDL = low-density lipoprotein; P = phosphorus; RBC = red blood cell count; WBC = white blood cell count.

Table S3. Univariable Logistic Regression Analysis of Factors Associated with Glaucoma

Variable	Odds Ratio	95% CI	*p* Value
**Demographics**			
Age, per year	1.06	1.02–1.11	0.005
Sex, male vs. female	2.12	1.63–2.77	<0.001
**Medical condition**			
Hypertension, yes vs. no	1.72	1.21–2.44	0.002
Diabetes mellitus, yes vs. no	1.01	0.50–2.06	0.97
Hyperlipidemia, yes vs. no	1.25	0.90–1.75	0.19
Heart disease, yes vs. no	1.26	0.52–3.09	0.61
Metabolic syndrome, yes vs. no	1.36	0.98–1.89	0.07
**Smoking**			
No (reference)	–		
Past	1.5	1.10–2.05	0.011
Current	1.15	0.86–1.55	0.34
**Drinking**			
No (reference)	–		
Past	1.28	0.62–2.67	0.50
Current	1.57	1.11–2.24	0.012
**Household income**			
<5 million KRW/month (reference)	–		
≥5 million KRW/month	1.44	1.11–1.87	0.06
**Education**			
< College (reference)	–		
≥ College	1.69	1.14–2.51	0.09
**Anthropometric**			
BMI (kg/m²)			
<25 (reference)	–		
≥25	1.14	0.86–1.50	0.36
WC (cm)			
Normal (reference)	–		
Abnormal	1.21	0.90–1.62	0.21

Blood pressure			
SBP, per mm Hg	1.02	1.01–1.03	<0.001
DBP, per mm Hg	1.02	1.01–1.03	<0.001
**Ocular characteristics**			
IOP, per mm Hg	1.17	1.12–1.22	<0.001
Retinal arteriosclerosis			
Absent (Grade 0, reference)	–		
Present (Grade ≥ 1)	6.49	4.87–8.65	<0.001
**Blood test**			
Glucose, per mg/dL	1	0.99–1.01	0.61
HbA1c, per %	0.9	0.69–1.17	0.41
Ca, per mg/dL	1.35	0.98–1.87	0.07
P, per mg/dL	0.82	0.62–1.06	0.13
BUN, per mg/dL	1.01	0.97–1.05	0.76
Creatinine, per mg/dL	3.47	1.89–6.39	<0.001
AST, per U/L	1	0.99–1.01	0.82
ALT, per U/L	1	1.00–1.00	0.46
ALP, per IU/L	1	1.00–1.01	0.16
GGT, per U/L	1	1.00–1.00	0.22
Total bilirubin, per mg/dL	1.02	0.89–1.16	0.79
Uric acid, per mg/dL	1.12	1.05–1.20	<0.001
Total cholesterol, per 10 mg/dL	1.01	1.00–1.01	0.002
LDL cholesterol, per 10 mg/dL	1.01	1.00–1.01	<0.001
HDL cholesterol, per 10 mg/dL	0.98	0.97–0.99	<0.001
Triglycerides, per 10 mg/dL	1	1.00–1.00	0.013
Total protein, per g/dL	0.89	0.65–1.21	0.46
Albumin, per g/dL	1.82	1.10–3.00	0.019
Hs-CRP, per mg/dL	1.20	0.86-1.68	0.28
Blood concentration of			
WBC, per 10^3/μL	1.01	0.97–1.04	0.70
RBC, per 10^6/μL	1.93	1.50–2.49	<0.001
Hematocrit, per 1%	1.06	1.03–1.09	<0.001
HBsAg positivity	0.97	0.51–1.83	0.94
HCV-Ab positivity	0.84	0.12–6.09	0.87

Univariable odds ratios and 95% confidence intervals were calculated using logistic regression analyses. Continuous variables were analyzed per unit increase, and categorical variables were analyzed relative to the indicated reference category.
ALP = alkaline phosphatase; ALT = alanine aminotransferase; AST = aspartate aminotransferase; BUN = blood urea nitrogen; Ca = calcium; CI = confidence interval; DBP = diastolic blood pressure; GGT = gamma-glutamyl transferase; HbA1c = hemoglobin A1c; HBsAg = hepatitis B surface antigen; HCV-Ab = hepatitis C virus antibody; HDL = high-density lipoprotein; Hs-CRP = high-sensitivity C-reactive protein; LDL = low-density lipoprotein; P = phosphorus; RBC = red blood cell count; SBP = systolic blood pressure; WBC = white blood cell count.
